# Complete Genome Sequence of Avian Pathogenic Escherichia coli Strain APEC O2-211

**DOI:** 10.1128/MRA.01046-18

**Published:** 2018-09-27

**Authors:** Daniel W. Nielsen, Paul Mangiamele, Nicole Ricker, Nicolle L. Barbieri, Heather K. Allen, Lisa K. Nolan, Catherine M. Logue

**Affiliations:** a Department of Veterinary Microbiology and Preventive Medicine, College of Veterinary Medicine, Iowa State University, Ames, Iowa, USA; b Roche Sequencing Solutions, Belmont, California, USA; c Food Safety and Enteric Pathogens Research Unit, National Animal Disease Center, ARS-USDA, Ames, Iowa, USA; d Department of Population Health, College of Veterinary Medicine, University of Georgia, Athens, Georgia, USA; e Department of Infectious Diseases, College of Veterinary Medicine, University of Georgia, Athens, Georgia, USA; University of Arizona

## Abstract

Avian pathogenic Escherichia coli (APEC) is the causative agent of colibacillosis, a disease that affects poultry production worldwide and leads to multimillion-dollar losses annually. Here, we report the genome sequence of APEC O2-211, a sequence type 117 (ST117) strain isolated from a diseased chicken.

## ANNOUNCEMENT

Avian pathogenic Escherichia coli (APEC) O2-211 is a serogroup O2, sequence type 117 strain isolated from the air sac of a chicken clinically diagnosed with colibacillosis (1). APEC O2-211 is also present in the literature as APEC 211 or isolate number 820905 (1–3).

APEC O2-211 was stored at −80°C in glycerol prior to sequencing, and the isolate was cultured on MacConkey agar. Next, a single colony was picked and grown in Luria-Bertani broth at 37°C. DNA was extracted using a DNeasy blood and tissue Genomic-tip kit (Qiagen, Hilden, Germany) for Pacific Bioscience (PacBio, Menlo Park, CA) sequencing and a ChargeSwitch genomic DNA (gDNA) mini bacteria kit (Life Technologies, Carlsbad, CA) for Illumina sequencing. DNA yields were quantified using a Qubit fluorometer double-stranded DNA (dsDNA) high-sensitivity (HS) assay kit (Life Technologies). The genomic library for MiSeq sequencing was prepared with a Nextera Flex kit (Illumina, San Diego, CA), and a SMRTbell kit (Pacific Biosciences, Menlo Park, CA) was used to prepare the genomic library for PacBio sequencing with BluePippin (Sage Science, Beverly, MA) size selection to target a library size of 10 kb.

Genomic sequencing was performed on RS II (PacBio, Menlo Park, CA) and MiSeq (Illumina, San Diego, CA) instruments. Two single-molecule real-time (SMRT) cells were used for PacBio sequencing. The Illumina data were trimmed using Trimmomatic v. 0.36 (4) and assembled *de novo* using the SPAdes assembler v. 3.10.1 (5). The PacBio data were assembled with Canu v. 1.5 (6). Next, the PacBio assembly was circularized in Geneious v. 10.2.3 (7) and further corrected and polished once with paired Illumina reads utilizing Pilon v. 1.22 (Broad Institute) (8). Potential contigs with plasmids were identified from both sets of sequencing data with the PlasmidFinder database (9) in ABRicate (https://github.com/tseemann/abricate). Protein-encoding genes and tRNA-carrying genes were assessed via Prokka v. 1.13 (10). A complete workflow and assembly report can be found at http://github.com/nielsend/genomeassembly.

The genome of APEC O2-211 consists of a single chromosome, one large plasmid, and two small plasmids. The chromosome consists of 5,114,241 bp and has a 50.6% GC content. It encodes 89 tRNAs and contains 4,783 coding sequences. The large plasmid, pAPECO2-211A-ColV, is a hybrid IncFIB/IncFIC plasmid. It consists of 197,773 bp with 215 coding sequences and has a 49.1% GC content. The two small plasmids, pAPECO2-211B and pAPECO2-211C, consist of 4,231 and 2,096 bp, respectively. Genomic comparisons of APEC O2-211 with other extraintestinal pathogenic E. coli (ExPEC) are ongoing.

### Data availability.

The chromosomes and plasmids have been deposited in GenBank under the accession numbers CP006834, CP030791, CP030792, and CP030793. The PacBio and Illumina reads are available in the NCBI Sequence Read Archive (accession number SRP158042).
